# Direct Reprogramming of Huntington’s Disease Patient Fibroblasts into Neuron-Like Cells Leads to Abnormal Neurite Outgrowth, Increased Cell Death, and Aggregate Formation

**DOI:** 10.1371/journal.pone.0109621

**Published:** 2014-10-02

**Authors:** Yanying Liu, Yuanchao Xue, Samantha Ridley, Dong Zhang, Khosrow Rezvani, Xiang-Dong Fu, Hongmin Wang

**Affiliations:** 1 Division of Basic Biomedical Sciences, University of South Dakota Sanford School of Medicine, Vermillion, South Dakota, United States of America; 2 Department of Cellular and Molecular Medicine, University of California San Diego, The Palade Laboratories Room 231, La Jolla, California, United States of America; University of Maryland School of Pharmacy, United States of America

## Abstract

Recent advances in trans-differentiation of one type cell to another have made it possible to directly convert Huntington’s disease (HD) patient fibroblasts into neurons by modulation of cell-lineage-specific transcription factors or RNA processing. However, this possibility has not been examined. Here, we demonstrate that HD patient-derived fibroblasts can be directly trans-differentiated into neuron-like cells by knockdown of the expression of a single gene encoding the polypyrimidine-tract-binding protein. The directly converted HD neuron-like cells were positive in expression of Tuj1, NeuN, DARPP-32, and γ-aminobutyric acid and exhibited neuritic breakdown, abnormal neuritic branching, increased cell death, and aggregation of mutant huntingtin. These observations indicate that the neuron-like cells directly converted from HD patient fibroblasts recapitulate the major aspects of neuropathological characteristics of HD and thus provide an additional model for understanding the disorder and validation of therapeutic reagents.

## Introduction

Huntington’s disease (HD) is a progressive neurodegenerative disorder caused by expansion of polyglutamine (polyQ) repeats in the N-terminus of the huntingtin (Htt) protein [1], [2]. The disease is neuropathologically characterized by neuronal loss in the striatum and cortex and formation of protein aggregates (inclusions), resulting in motor and behavioral dysfunction [3]. To understand the pathogenesis of HD, a number of HD cell models have been created and applied in many studies over the last two decades [4], [5]. Although these HD cells exhibit at least some of the pathological features of HD, most of them do not express full-length human mutant Htt and neuronal markers and thus are not ideal for modeling HD. Induced pluripotent stem cells from HD patient or animal fibroblasts provide a new model for studying HD [6]–[9]. However, the neuronal induction process is usually time-consuming and tedious. Recently, trans-differentiation of one type cell to another has been made it possible to directly convert HD patient fibroblasts into neuron-like cells by modulation of cell-lineage-specific transcription factors or RNA processing [10]–[12]. However, it remains unknown whether HD patient-derived fibroblasts can be directly reprogrammed into the neuron-like cells that reproduce the major aspect of HD pathological features.

The polypyrimidine-tract-binding (PTB) is an RNA-binding protein that regulates RNA splicing, stability, and localization [13]. During neuronal differentiation, the expression of PTB is switched to its neuronal homolog, nPTB [14]. Forced expression of PTB blocks neuronal differentiation [15], whereas knockdown of PTB expression by PTB-RNA interactions dramatically promotes conversion of diverse cell types into neurons [12], [16]. Here, we demonstrate that following PTB knockdown, HD patient-derived fibroblasts can be directly reprogrammed to neuron-like cells that exhibit the major HD pathological characteristics.

## Materials and Methods

### Ethics statement

The following cell lines were obtained from the NIGMS Human Genetic Cell Repository at the Coriell Institute for Medical Research: AG07095, GM04281, and GM05539. The Coriell Institute and ATCC maintain the written consent forms and privacy of the donors of the fibroblast samples, and the authors had no contact or interaction with the donors. All human fibroblast cells and protocols in the present study were carried out in accordance with the guidelines approved by the University of South Dakota Institutional Review Board.

### Cell culture, preparation and infection of PTB1 small-hairpin (sh) RNA lentiviral particles

Human fibroblasts were maintained in DMEM supplemented with 10% defined FBS, non-essential amino acids, Glutamax, β-mercaptoethanol and 100 ng/mL bFGF at 37°C, 5% CO_2_. The CAG repeat number information in the htt gene was obtained from Coriell and confirmed by PCR using a PCR kit (Genelink).

Preparation of lentiviral particles of the shRNAs against human PTB1 and infection of fibroblasts were performed as previously described [12]. Sixteen hours after the shRNA treatment, the cells were selected either with 2 µg/ml puromycin or 100 ng/µl of hygromycin B for 48 h. Selected cells were switched into N3 medium (DMEM/F12, 25 µg/ml insulin, 50 µg/ml human transferrin, 30 nM sodium selenite, 20 nM progesterone, and 100 nM putrescine) supplemented with FGF2 (10 ng/ml) for 3 days and then switched to N3 medium for 10 days. Finally, cells were maintained in N3 medium supplemented with BDNF, GDNF, NT3 and CNTF as previously described [12] until being used for different analyses.

### Immunocytochemistry and fluorescence and confocal microscopy

Immunocytochemical staining was performed according to our previously described method [17]. Primary antibodies used include anti-Tuj1 (1∶100, Millipore), anti-NeuN (1∶100, Millipore), anti-gamma amminobutyric acid (GABA) (1∶1000, Millipore), anti-DARPP-32 (1∶50, Santa Cruz Biotechnology), and Htt EM48 (1∶100, Millipore). Nuclei were stained with Hoechst 33342 (Life Technologies) as previously described [18], [19]. Images were acquired with a Carl Zeiss fluorescence microscope equipped with the Axiocam HRM ZEISS camera and AxioVision software. For cells stained with Htt EM48 antibody, images were captured with an Olympus confocal laser scanning microscope equipped with an argon laser and two HeNe lasers and FluoView 1000 software.

### Cell counting

If a Tuj1-positive cell had lost all neurites or showed neurite breakdown, the cell would be treated as a cell with neuritic degeneration. Tuj1-positive cells with less than 20 µm in length of neurites or showing apparent thin neurites were regarded as cells with abnormal neuritic branching. GABA-positive cells with neurite breakdown and/or shrunken nuclei/cell bodies were counted as degenerated cells. If a cell had a nucleus containing one or more Htt aggregates, the cell would be counted as the positive for nuclear inclusion. If a cell contains aggregate(s) in the non-nuclear soma region or inside a neurite, the cell would be counted as the positive for non-nuclear (soma/neuropil) aggregate. At least 50 cells were counted in each experiment group and three independent experiments were performed.

### Detection of apoptotic cells

Apoptotic cell death was examined as previously described [20] by utilizing a TUNEL (terminal deoxynucleotidyl transferase dUTP nick end labeling, TUNEL) based apoptosis detection kit (Millipore). Stained cells were observed with a fluorescence microscope. Apoptotic cell rate was calculated as follows: apoptotic (or TUNEL positively stained cell) rate (%) = number of TUNELpositively stained cells/number of total cells (assessed by Hoechst 33342 staining)**×**100%.

### Statistical analysis

Statistical comparisons between two groups were evaluated using two-tailed student’s t test. P<0.05 was regarded as statistically significant.

## Results and Discussion

**Figure 1:**
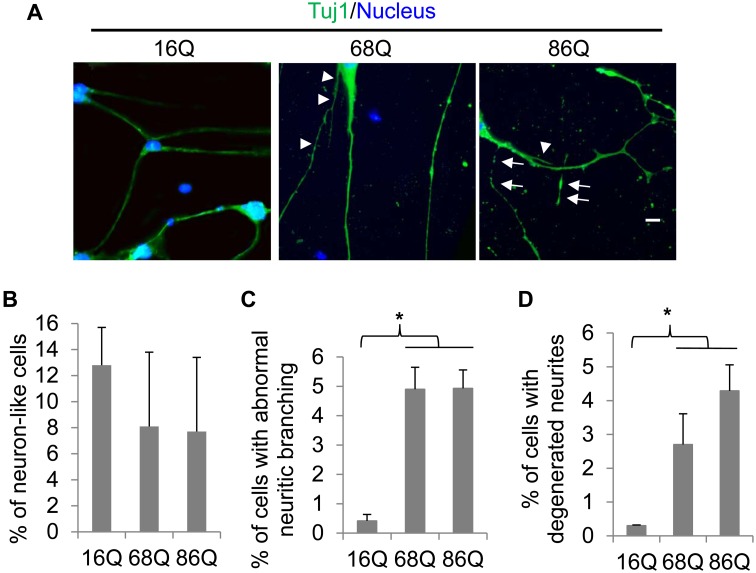
Direct conversion of HD patient fibroblasts into neuron-like cells. Human fibroblasts derived from a normal individual (16Q) or HD patients (68Q and 86Q) were infected with lentiviral shRNAs against human PTB. Nineteen days following the treatment, cells were used for analyses. *(* ***A*** *)* Immunostaining of the trans-differentiated cells with a neuron-specific marker, Tuj1, antibody. Arrow heads indicate thin or short neurites and arrows show broken neurites. Scale bar, 10 µm. Cell counting results showing the percentage of cells with neuron-like morphology positive in Tuj1 staining *(* ***B*** *)* ***,*** with abnormal neurites *(* ***C*** *)* ***,*** or with degenerated (breakdown) neurites *(* ***D*** *)* ***.*** Data are shown as mean ± SD; n = 3. *p<0.05.

To reprogram the fibroblasts derived from HD patients into neuron-like cells, we employed a recently described method to knock down PTB protein [12] by infecting HD patient fibroblasts expressing Htt containing either 16Q, 68Q, or 86Q with lentiviral shRNAs against human PTB. Nineteen days following PTB knockdown, the cells exhibited a typical neuron-like morphology and showed positive immunoreactivity with Tuj1, a neuron-specific cytoskeleton protein present in newly generated immature postmitotic neurons and differentiated neurons [20], [21] (Fig. 1A). Cell counting showed that the HD patient-derived fibroblasts did not significantly differ from the normal fibroblasts in the capability of conversion to Tuj1-positive neuron-like cells (Fig. 1B). Those undifferentiated cells did not show Tuj1 staining and only showed the nuclear staining (Fig. 1A). The Tuj1-positive cells converted from HD patients (referred to as 68Q and 86Q, respectively) showed different neuritic morphology from the cells derived from a normal individual (16Q). The normal fibroblast-converted cells extended from one to several relatively thick neurites directly from the cell body (Fig. 1A, left panel). In addition to the thick neurites, however, the HD fibroblasts-derived cells frequently grew out thin neurites either directly from their cell bodies or from thick neurites (Figs. 1A, middle and right panels, pointed by arrow heads). Interestingly, some of the neurites derived from HD neuron-like cells were broken down and degenerated into small fragments positive in Tuj1 staining (Fig. 1A, pointed by arrows). Additionally, cell counting results indicated that more HD neuron-like cells (68Q and 86Q) exhibited abnormal neuritic branching (Fig. 1C) and neuritic breakdown (Fig. 1D) than the wild-type of neuron-like cells (16Q). These results indicate that the trans-differentiation of HD patient’s fibroblasts into neuron-like cells leads to abnormal neuritic branching and degeneration.

**Figure 2:**
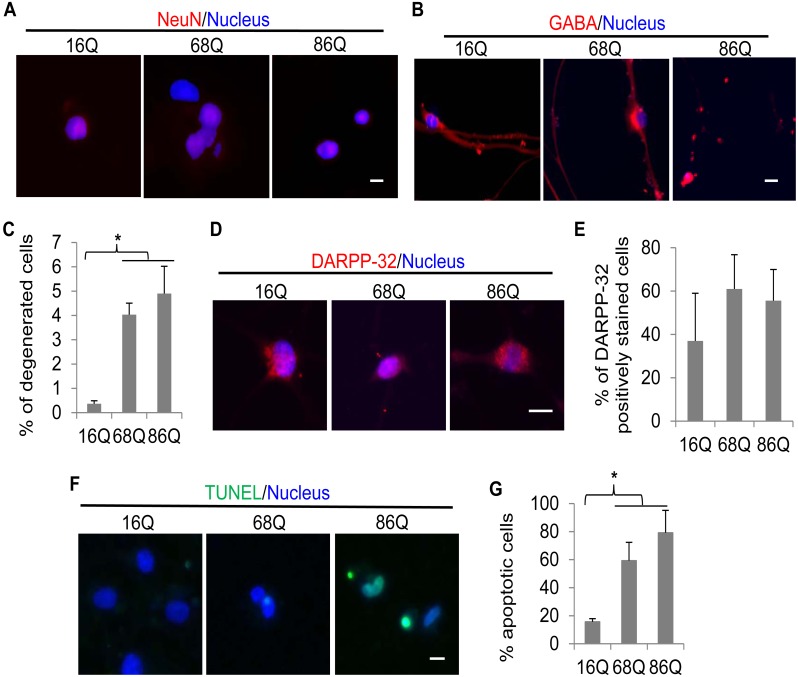
The directly converted HD neuron-like cells are positive in NeuN, GABA, and DARPP-32 expressions and show progressive cell death. Immunostaining of the trans-differentiated cells with a NeuN *(* ***A*** *)* or GABA *(* ***B*** *)* antibody at nineteen days following PTB knockdown. Scale bar, 10 µm. *(* ***C*** *)* Quantification results showing the percentage of degenerated GABA-positive cells derived from the three types of fibroblasts at nineteen days. *(* ***D*** *)* Immunostaining of the trans-differentiated cells with a DARPP-32 antibody at thirty days. Scale bar, 10 µm. *(* ***E*** *)* Graph showing the percentage of DARPP-32 positive cells at thirty days following PTB knockdown. *(* ***F*** *)* TUNEL staining of the converted cells at thirty days following the reprogramming. Scale bar, 10 µm. *(* ***G*** *)* Graph showing the percentage of apoptotic cells assessed by TUNEL staining shown in *(* ***F*** *).* All quantitative data are shown as mean ± SD; n = 3 for each group of cells. *p<0.001.

As the neuronal nuclear antigen (NeuN) is a nuclear protein widely expressed in the mature postmitotic neurons, it has been commonly used as a neuron-specific marker for mature neurons [22]. We thus stained the cells with a NeuN specific antibody and found that at least 10% cells were positive in NeuN expression in each of the three converted cell types after nineteen days of the reprogramming (Fig. 2A). Since one major pathological feature of HD is selective loss of GABAergic neurons in the striatum [23], we next examined whether the trans-differentiated neuron-like cells express γ-aminobutyric acid (GABA), an inhibitory neurotransmitter. As shown in Fig. 2B, GABA was strongly expressed in both the normal and HD neuron-like cells nineteen days after shRNA knockdown of PTB. Compared to the normal fibroblast-derived GABA-positive cells, some HD GABA-positive cells showed degenerating neurites and shrunken cell bodies (Fig. 2B, right panel). Cell counting indicates that neurodegeneration was significantly more in the HD neuron-like cells than in the normal cells (Fig. 2C). Additionally, as degenerated neurons in HD striatum are DARPP-32 positive cells [23], we examined whether the trans-differentiated neuron-like cells are also positively stained with the protein. As shown in Figs. 2D and 2E, thirty days following the reprogramming, many cells expressed DARPP-32. At this time point, however, degenerated cells were dramatically increased to 59% and 79% in the 68Q and 86Q HD cells, respectively (Figs. 2F, 2G). Taken together, these data suggest that the HD patient fibroblasts can be trans-differentiated to GABA and DARPP-32-positive neuron-like cells and the reprogramming triggers increased cell death in the HD fibroblast-derived cells.

**Figure 3:**
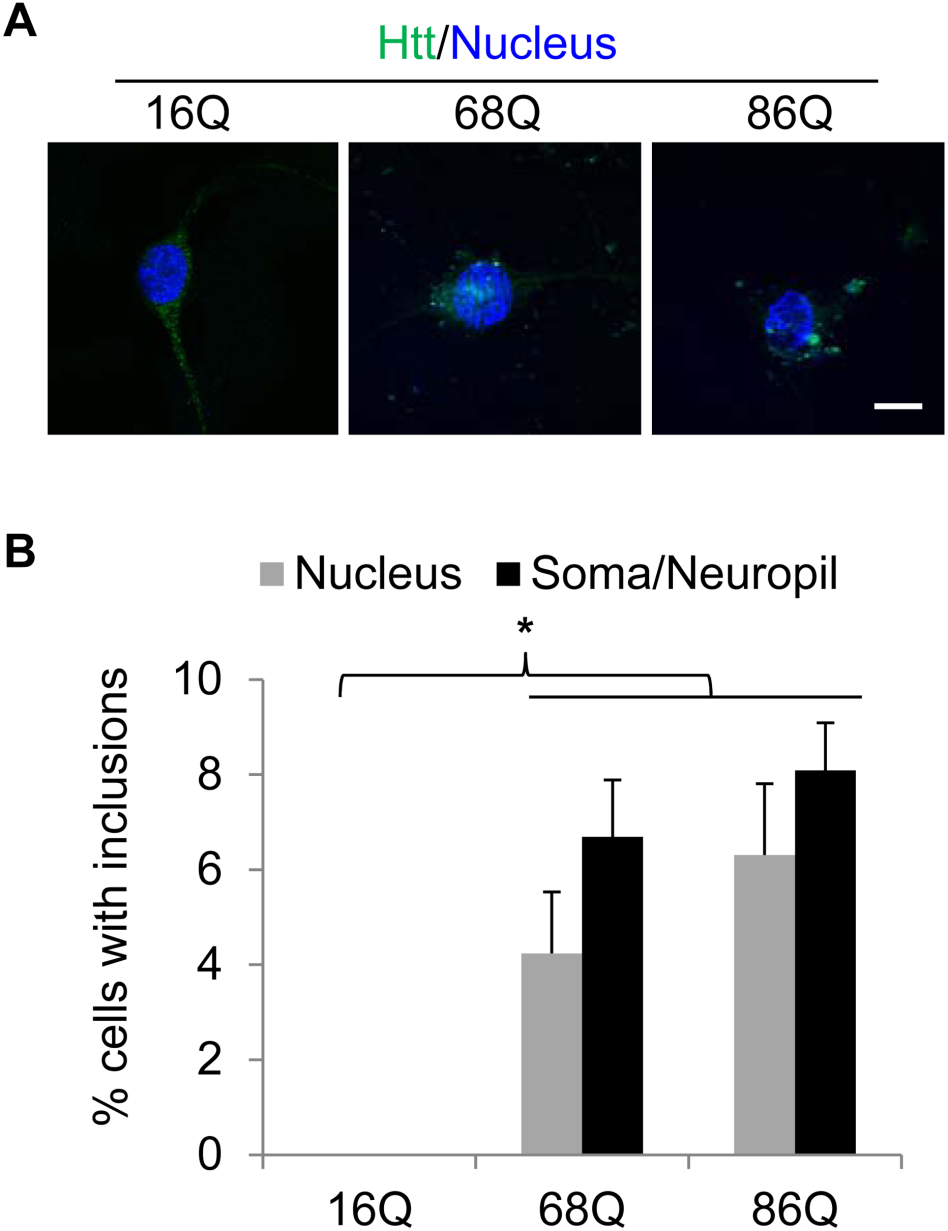
The directly converted HD neuron-like cells show Htt inclusions at nineteen days following PTB knockdown. *(* ***A*** *)* Immunostaining of the trans-differentiated cells with the EM48 antibody indicates the presence of Htt inclusions in the HD cells. Scale bar, 10 µm. *(* ***B*** *)* Cell counting results showing the percentage of cells with aggregates in the nucleus or non-nuclear regions (soma and/or neuropil). Data are shown as mean ± SD; *p<0.001. n = 3.

We next examined whether trans-differentiation of HD patient fibroblasts to neuron-like cells leads to mutant Htt aggregation. We therefore immunostained the three types of converted neuron-like cells with the well-documented EM48 Htt antibody, which selectively binds to the toxic N-terminal fragment of the mutant Htt protein [24], and then assessed the cells positive with inclusions in the nucleus, soma, and neuropil by confocal microscopy. There was no EM48-positive nuclear inclusion in the neuron-like cells trans-converted from the normal fibroblasts, whereas most of the neuron-like cells converted from the two types of HD fibroblasts contained Htt inclusions in their nuclei and non-nuclear regions (soma and neuropils) (Figs. 3A, 3B). These results indicate that the mutant Htt proteins preferentially form aggregates in both the nucleus and non-nuclear regions upon conversion to neuron-like cells.

One interesting observation from this research is that direct conversion of HD patient fibroblasts to neuron-like cells leads to abnormal neurite outgrowth and branching, characterized by frequently short or thin neurite outgrowth. This is in accordance with a previous *in vivo* study, in which abnormal dendritic arbors and increased dendritic branching in spiny striatal neurons were identified in post-mortem HD patients’ brain sections [25]. Although it remains unclear why the HD neuron-like cells selectively exhibit this dysmorphic alteration, mutant Htt-caused intracellular trafficking dysfunction may be, at least partially, responsible for abnormal neurite outgrowth and branching [26]. Additionally, mutant Htt also impairs mitochondrial integrity [27] and disrupts production and trafficking of neurotrophic factors [28], which may also affect neurite outgrowth and branching. As a further direction, it is interesting to explore the biological significance underlying this dysmorphic alteration. In addition to showing increased cell death, the directly trans-converted HD cells also form aggregates not only in the nucleus but also in non-nuclear regions such as neuropils, which is in accordance with previous *in vivo* studies using HD patient brain tissues [29]. Thus, the directly converted neuron-like cells from HD fibroblasts provide a reliable model for studying pathogenic mechanisms of HD and may be a useful tool for validation of therapeutic target or drugs in the future.

## References

[1] The-Huntington-disease-collaborative-research-group (1993) A novel gene containing a trinucleotide repeat that is expanded and unstable on Huntington’s disease chromosomes. The Huntington’s Disease Collaborative Research Group. Cell 72: 971–983. 8458085 10.1016/0092-8674(93)90585-e

[2] Li SH, Li XJ (2004) Huntingtin and its role in neuronal degeneration. Neuroscientist 10: 467–475. 15359012 10.1177/1073858404266777

[3] Reiner A, Albin RL, Anderson KD, D’Amato CJ, Penney JB, et al (1988) Differential loss of striatal projection neurons in Huntington disease. Proc Natl Acad Sci U S A 85: 5733–5737. 2456581 10.1073/pnas.85.15.5733 PMC281835

[4] Wyttenbach A, Swartz J, Kita H, Thykjaer T, Carmichael J, et al (2001) Polyglutamine expansions cause decreased CRE-mediated transcription and early gene expression changes prior to cell death in an inducible cell model of Huntington’s disease. Hum Mol Genet 10: 1829–1845. 11532992 10.1093/hmg/10.17.1829

[5] Sipione S, Cattaneo E (2001) Modeling Huntington’s disease in cells, flies, and mice. Mol Neurobiol 23: 21–51. 11642542 10.1385/MN:23:1:21

[6] Park IH, Arora N, Huo H, Maherali N, Ahfeldt T, et al (2008) Disease-specific induced pluripotent stem cells. Cell 134: 877–886. 18691744 10.1016/j.cell.2008.07.041 PMC2633781

[7] Chan AW, Cheng PH, Neumann A, Yang JJ (2010) Reprogramming Huntington monkey skin cells into pluripotent stem cells. Cell Reprogram 12: 509–517. 20936902 10.1089/cell.2010.0019 PMC2993046

[8] The-HD-iPSC-Consortium (2012) Induced pluripotent stem cells from patients with Huntington’s disease show CAG-repeat-expansion-associated phenotypes. Cell Stem Cell 11: 264–278. 22748968 10.1016/j.stem.2012.04.027 PMC3804072

[9] Kaye JA, Finkbeiner S (2013) Modeling Huntington’s disease with induced pluripotent stem cells. Mol Cell Neurosci 56: 50–64. 23459227 10.1016/j.mcn.2013.02.005 PMC3791169

[10] Ambasudhan R, Talantova M, Coleman R, Yuan X, Zhu S, et al (2011) Direct reprogramming of adult human fibroblasts to functional neurons under defined conditions. Cell Stem Cell 9: 113–118. 21802386 10.1016/j.stem.2011.07.002 PMC4567246

[11] Vierbuchen T, Ostermeier A, Pang ZP, Kokubu Y, Sudhof TC, et al (2010) Direct conversion of fibroblasts to functional neurons by defined factors. Nature 463: 1035–1041. 20107439 10.1038/nature08797 PMC2829121

[12] Xue Y, Ouyang K, Huang J, Zhou Y, Ouyang H, et al (2013) Direct conversion of fibroblasts to neurons by reprogramming PTB-regulated microRNA circuits. Cell 152: 82–96. 23313552 10.1016/j.cell.2012.11.045 PMC3552026

[13] Valcarcel J, Gebauer F (1997) Post-transcriptional regulation: the dawn of PTB. Curr Biol 7: R705–708. 9382788 10.1016/s0960-9822(06)00361-7

[14] Boutz PL, Stoilov P, Li Q, Lin CH, Chawla G, et al (2007) A post-transcriptional regulatory switch in polypyrimidine tract-binding proteins reprograms alternative splicing in developing neurons. Genes Dev 21: 1636–1652. 17606642 10.1101/gad.1558107 PMC1899473

[15] Makeyev EV, Zhang J, Carrasco MA, Maniatis T (2007) The MicroRNA miR-124 promotes neuronal differentiation by triggering brain-specific alternative pre-mRNA splicing. Mol Cell 27: 435–448. 17679093 10.1016/j.molcel.2007.07.015 PMC3139456

[16] Xue Y, Zhou Y, Wu T, Zhu T, Ji X, et al (2009) Genome-wide analysis of PTB-RNA interactions reveals a strategy used by the general splicing repressor to modulate exon inclusion or skipping. Mol Cell 36: 996–1006. 20064465 10.1016/j.molcel.2009.12.003 PMC2807993

[17] Dong G, Ferguson JM, Duling AJ, Nicholas RG, Zhang D, et al (2011) Modeling pathogenesis of Huntington’s disease with inducible neuroprogenitor cells. Cell Mol Neurobiol 31: 737–747. 21452052 10.1007/s10571-011-9679-0 PMC3724999

[18] Dong G, Gross K, Qiao F, Ferguson J, Callegari EA, et al (2012) Calretinin interacts with huntingtin and reduces mutant huntingtin-caused cytotoxicity. J Neurochem 123: 437–446. 22891683 10.1111/j.1471-4159.2012.07919.x

[19] Dong G, Callegari EA, Gloeckner CJ, Ueffing M, Wang H (2012) Prothymosin-alpha interacts with mutant huntingtin and suppresses its cytotoxicity in cell culture. J Biol Chem 287: 1279–1289. 22110140 10.1074/jbc.M111.294280 PMC3256907

[20] Menezes JR, Luskin MB (1994) Expression of neuron-specific tubulin defines a novel population in the proliferative layers of the developing telencephalon. J Neurosci 14: 5399–5416. 8083744 10.1523/JNEUROSCI.14-09-05399.1994 PMC6577108

[21] von Bohlen Und Halbach O (2007) Immunohistological markers for staging neurogenesis in adult hippocampus. Cell Tissue Res 329: 409–420. 17541643 10.1007/s00441-007-0432-4

[22] Lavezzi AM, Corna MF, Matturri L (2013) Neuronal nuclear antigen (NeuN): a useful marker of neuronal immaturity in sudden unexplained perinatal death. J Neurol Sci 329: 45–50. 23570982 10.1016/j.jns.2013.03.012

[23] Vonsattel JP, DiFiglia M (1998) Huntington disease. J Neuropathol Exp Neurol 57: 369–384. 9596408 10.1097/00005072-199805000-00001

[24] Zhou H, Cao F, Wang Z, Yu ZX, Nguyen HP, et al (2003) Huntingtin forms toxic NH2-terminal fragment complexes that are promoted by the age-dependent decrease in proteasome activity. J Cell Biol 163: 109–118. 14557250 10.1083/jcb.200306038 PMC2173440

[25] Ferrante RJ, Kowall NW, Richardson EP Jr (1991) Proliferative and degenerative changes in striatal spiny neurons in Huntington’s disease: a combined study using the section-Golgi method and calbindin D28k immunocytochemistry. J Neurosci 11: 3877–3887. 1836019 10.1523/JNEUROSCI.11-12-03877.1991 PMC6575286

[26] Rong J, McGuire JR, Fang ZH, Sheng G, Shin JY, et al (2006) Regulation of intracellular trafficking of huntingtin-associated protein-1 is critical for TrkA protein levels and neurite outgrowth. J Neurosci 26: 6019–6030. 16738245 10.1523/JNEUROSCI.1251-06.2006 PMC6675209

[27] Wang H, Lim PJ, Karbowski M, Monteiro MJ (2009) Effects of overexpression of huntingtin proteins on mitochondrial integrity. Hum Mol Genet 18: 737–752. 19039036 10.1093/hmg/ddn404 PMC2722218

[28] Ferrer I, Goutan E, Marin C, Rey MJ, Ribalta T (2000) Brain-derived neurotrophic factor in Huntington disease. Brain Res 866: 257–261. 10825501 10.1016/s0006-8993(00)02237-x

[29] Gutekunst CA, Li SH, Yi H, Mulroy JS, Kuemmerle S, et al (1999) Nuclear and neuropil aggregates in Huntington’s disease: relationship to neuropathology. J Neurosci 19: 2522–2534. 10087066 10.1523/JNEUROSCI.19-07-02522.1999 PMC6786077

